# Dyads use heuristics to minimise time costs during joint action

**DOI:** 10.1038/s41598-025-98394-1

**Published:** 2025-05-07

**Authors:** Shaheed Azaad, Natalie Sebanz

**Affiliations:** Department of Cognitive Science, Central European University, Vienna, Austria

**Keywords:** Joint action, Action planning, Efficiency, Coordination, Human behaviour, Psychology

## Abstract

Research on joint action planning has demonstrated that, when acting with others, individuals will choose more effortful actions if it improves *co-efficiency* by reducing the overall effort exerted by the group. However, because these studies use actions for which time and effort costs are confounded, it is unclear which costs participants sought to minimise and what processes underlie decision-making about individual contributions to joint actions. Across three experiments, we tested (1) whether dyads aim to minimise effort vs. time costs (Experiments 1–2) and (2) whether individuals choose actions based on a rational, deliberative process or a heuristic (Experiment 3). Data from a joint object-dragging computer task revealed that participants chose to drag objects to the closer of two goals even when it required more effort from the dyad (Experiments 1–2). Participants preferred closer goals when time and effort costs were equal (Experiment 3a), but only when the objects’ locations were salient (Experiment 3b). Together, these results suggest that: (1) individuals minimise time, rather than effort, when sharing object-moving tasks, and (2) they do so – at least in part - by using distance as a heuristic. Our findings also replicate and extend earlier work showing that people prefer acting together versus alone, even when this is less efficient.

## Introduction

We can often achieve our goals through more than one course of action - all else being equal, we tend to choose the least effortful^1^. People typically prefer direct over indirect paths^2^, actions that require less force^3^, and to offload cognitively demanding tasks^4^. A topical question in joint action research is how we apply cost-minimisation strategies when working with others. So far, results have shown that people reliably choose actions that require the least effort for the dyad, even if it means more effort for either themselves or their partner. In other words, people maximise *co-efficiency* over individual efficiency. While people’s tendency to act co-efficiently is well-established, less is known about the specific processes by which dyads choose less effortful courses of action. Across three experiments, we tested whether, when choosing an action, dyads (1) explicitly seek to minimise overall effort and (2) use heuristics or engage in more elaborate rational decision-making processes to determine (co-)efficiency.

## How do people achieve co-efficiency in joint action?

Joint action research has shown that we account for others’ effort costs when choosing a course of action. Santamaria and Rosenbaum^5^ conducted an observational study that recorded peoples’ tendency to hold a door open for those walking behind them. The study revealed that people were more likely to hold the door (1) the closer the follower(s) were and (2) when there were two followers rather than one. That is, door-holders were less likely to hold the door when it required more effort from them but also more likely when it saved two people the effort of opening the door. Together, these findings imply that individuals might choose courses of action that minimise group effort, even at their own cost.

Török et al.^6^ sought to directly test whether individuals would choose action plans that prioritise minimising overall - or dyadic - effort over individual effort. Pairs of participants completed a touchscreen computer task in which they needed to drag an object from its start location to a goal on the other side of the screen via one of two sub-goals. On each trial, a trial ‘leader’ would drag the ball away from themselves to one of two sub-goals at the centre of the screen. The second participant would then drag the object from the chosen sub-goal toward themselves to the final goal. Critically, a variable-length obstacle - around which participants would need to drag the object - determined the relative drag distances for each participant and the dyad. The key finding from this experiment was that the trial leader would choose the subgoal that resulted in an overall shorter path length for the dyad, even when it meant either they themselves or their partner would have to drag the object further than they would otherwise.

Based on their findings, Török et al. concluded that people use rational decision-making strategies to maximise efficiency (or minimise effort) for the dyad over the individual. Although subsequent studies have conceptually replicated Török et al. using different tasks^7,8^, all of the studies conducted so far leave open questions as to whether and, if so, how participants are employing a dyadic effort minimisation strategy. In previous studies, effort was always confounded with time costs. Since shorter paths require less effort and less time, participants could have sought to minimise their time on task rather than their exerted effort.

The possibility that participants might have prioritised time minimisation leads to a second limitation in Török et al. (2019) – that unlike effort costs, time costs for the dyad and the individual were the same. Since neither participant could move on to the subsequent trial until the second-acting participant completed their action, a strategy to minimise one’s own time or the dyad’s time would require the same action – to choose the shorter overall path. These two limitations allow for a markedly different alternative interpretation of Török et al. and similar studies – that people seek to minimise individual time and not combined effort.

Finally, while the findings on co-efficiency obtained so far align with the idea that co-actors engaged in a rational decision-making process that led them to choose co-efficient actions, they could also have relied on heuristics. Computing the effort costs of action alternatives requires perceiving and interpreting relevant cues. For instance, the size of an object might be a cue to how effortful it would be to lift, whereas its distance indicates the effort required to reach it. If such cues reliably indicate effort costs, people might use them as a heuristic to choose less costly actions.

## The present study

In sum, although prior work shows that people choose co-efficient action plans when coordinating, the processes by which they do so remain unclear. Across three experiments, we tested whether dyads (1) explicitly seek to minimise joint effort (Experiments 1 and 2) and (2) use heuristics (Experiment 3).

We asked pairs of participants to complete an object-dragging computer task in which they chose one of two goal locations to which to tap and drag objects. Our goal was to disentangle time and effort costs. We therefore varied the relative dragging distance of the goals and whether a goal required only one or both participant(s) to drag an object. Since participants acted simultaneously, this enabled us to disentangle time and effort costs – requiring both participants to act would double the dyad’s effort costs while keeping their time costs approximately the same. In Experiments 1 and 2, we presented trials in which the overall more efficient goal in terms of distance took longer to complete. In Experiment 3, we changed the response mode to a single tap that ‘teleported’ the objects to their goal. This way, time and effort costs remained constant as goal distance varied, enabling us to test whether people make decisions based on distance even when it is unrelated to efficiency.

We tested two sets of predictions: (1) If dyads seek to minimise overall effort in terms of distance, they should choose goals requiring shorter overall drag distances, even at a time cost. Conversely, if dyads minimise time, they should opt for faster goals even if they require more dragging overall (Experiments 1 and 2). (2) If dyads choose an action to explicitly minimise their costs, they should choose a goal randomly when the two goals require equal time and effort. Otherwise, if dyads decide on an action based on heuristics, they should choose the closer goal regardless (Experiment 3).

### Experiment 1

**Fig. 1:**
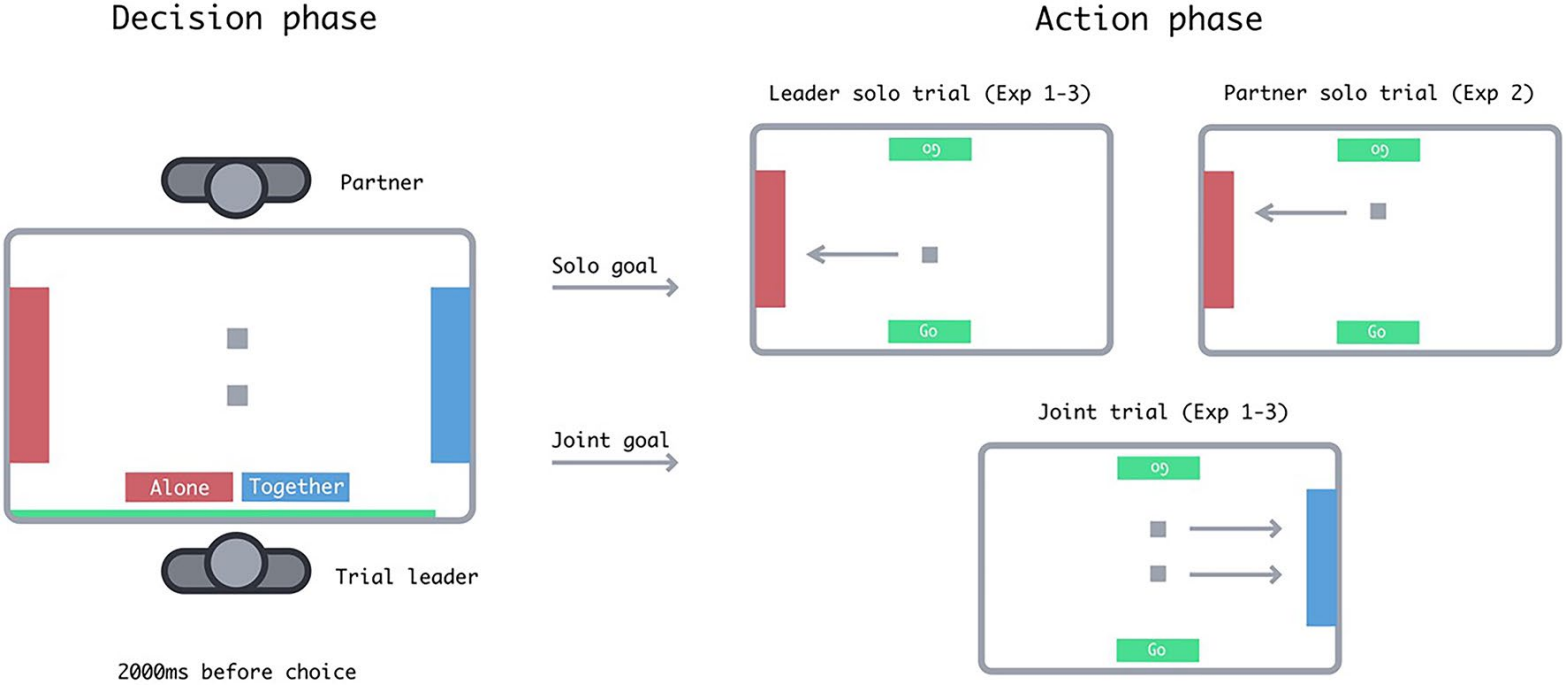
Trial structure. The green bar below the buttons in the decision phase indicated the time remaining until participants could choose a goal. Arrows in the action phase indicate the direction in which participants moved objects and were not visible in the experiment. Display elements are not to scale. Participants in Experiment 3b tapped the Green ‘Go’ indicator in the action phase to move objects.

In Experiment 1, pairs of participants completed an object-dragging computer task, in which they made per-trial decisions to drag objects to either (1) a joint goal requiring each participant to drag an object or (2) a solo goal that required one participant to drag a single object. Therefore, from a joint perspective, choosing a joint goal would require double the effort of a solo goal of identical distance. Since participants could drag simultaneously when acting together, choosing a joint goal did not similarly increase the time required to complete a trial. This enabled us to disentangle time and combined effort costs by having trials in which the less effortful goal was the slower option (Fig. 1).

**Table 1:** Overview of object starting-point conditions in experiments 1–3.

Condition	(1) Equi-distant	(2) Joint effortful	(3) Equal effort	(4) Solo effortful	(5) Solo goal closer	(6) Solo goal closer
Distance from joint goal	1/2	2/5	1/3	2/7	5/7	3/5
Goal requiring less dragging overall	Solo	Solo	Equal	Joint	Solo	Solo
Goal closer to the objects	Equal	Joint	Joint	Joint	Solo	Solo
Experiments	1–3	1–3^	1	1–3^	2–3	2–3

**Fig. 2:**
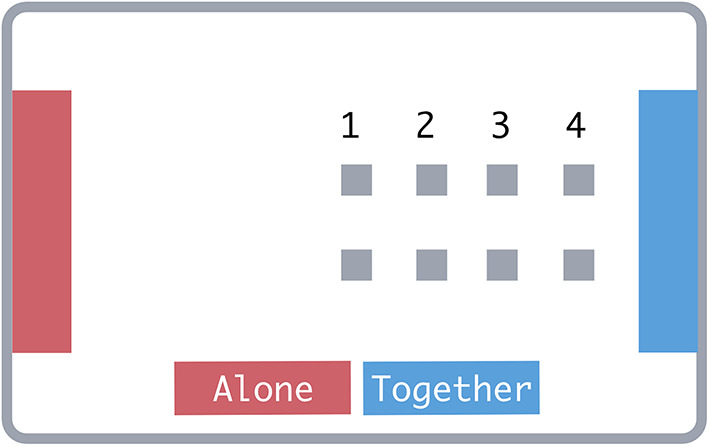
Object starting point conditions (display elements not to scale). (1) Equidistant, (2) Joint-effortful, (3) Equal effort, (4) Solo-effortful.

Condition numbers correspond to the object locations in Fig. 2. Distances are in units of the between-goal distance. These were the ‘joint goal closer’ conditions in Experiments 2–3.

Specifically, we presented participants with objects in four conditions (see Table 1 for an overview): (1) an *equidistant* condition where the objects were the same distance from either goal. In this case, both goals would require roughly the same time, but the joint goal would require double the overall dragging. (2) A *joint effortful* condition in which the joint goal was closer and thus faster with simultaneous dragging, but the combined dragging distance was still more than that of the solo alternative. (3) An *equal effort* condition in which the solo goal was double the distance of the joint goal. The combined drag distance for the joint goal was, therefore, the same as that for the solo goal while requiring around half the time to reach. (4) A *solo effortful* condition in which the solo goal was more than double the distance of the joint goal. In this case, the joint goal was faster and required less dragging overall than the solo goal.

We evaluated two sets of predictions. First, if dyads seek to minimise combined effort, they should choose (1) solo goals in the equidistant and joint effortful conditions, (2) joint goals in the solo effortful condition, and (3) randomly in the equal effort condition. Second, if dyads seek to minimise time, they will choose (1) a goal randomly in the equidistant condition and (2) joint goals in all other conditions.

## Method

### Participants

We based our power calculations on the barrier-length effect – which indicated participants’ sensitivity to the different effort ratios across trials – in Experiment 2 of Török et al. (2019). After fitting a binomial generalised mixed-effects model to participants’ choices, we ran power simulations using the *simr*^9^ package for R. Simulations revealed that with power = 0.90 alpha = 0.01 (for multiple comparisons), we would need just eight participants to detect between-condition differences of the magnitude reported in Török et al.

To ensure sufficient power to detect potentially smaller effects, we recruited 20 participants (12 female, 8 male, M_age_ = 25.5, SD _age_ = 5.4, 19 right-handed, 1 left-handed) from the Central European University (CEU PU)’s online participant pool. We obtained ethics approval for all experiments from the Psychological Research Ethics Board at CEU PU. We conducted all experiments in accordance with the Declaration of Helsinki. Participants in all experiments provided informed consent prior to participation.

### Procedure

Participants completed the object dragging task (presented using jsPsych^10^) on a 43-inch ProLite multi-touch display, facing each other and standing at the long edges of the screen. Each trial consisted of two stages– a decision stage and an action stage. Participants saw the trial layout in the decision stage before choosing a goal toward which to drag. The layout included two goal areas at either short edge of the screen. Each goal was 10% of the screen’s width and 75% of the screen’s height. Goals were either red or blue, depending on whether they required a solo or joint response. We counterbalanced response-colour mapping between subjects. Additionally, participants saw two square objects, each 2% of the screen’s height. These boxes’ locations varied on the x-axis (from the participants’ perspectives) across conditions. Boxes were centred on the y-axis with a 10% offset so that each box was closer to one of the two participants.

At the beginning of the decision phase, a timer and two initially-disabled option buttons appeared in front of the randomly-assigned trial leader. Buttons were labelled ‘Alone’ and ‘Together’, indicating which goal would require solo and joint dragging. After a 2000ms waiting period, as indicated by the timer, the buttons were enabled (indicated by switching from grey to colour), and the trial leader tapped one of them to begin the action stage. Buttons corresponded to the goals they represented both spatially and in colour.

The action phase commenced with a tone corresponding to whether the chosen goal required a joint or solo action. The option buttons and the non-chosen goal area disappeared from the screen, and if the leader chose a solo action, the object closer to their partner also disappeared. For solo goals, the leader would tap and drag the object from its starting point and into the goal area. For joint goals, participants simultaneously tapped and dragged the object closest to them into the goal area. A green ‘Go’ indicator appeared for the participant(s) required to act. We instructed participants that they did not need to synchronise or coordinate their drags. A tone indicated when the object was successfully placed into a goal area. The trial ended when the object(s) reached the goal.

Trials randomly appeared in one of four conditions with equal probability (Fig. 2; Table 1 for overview). In the *equidistant* condition, the objects were centred on the x-axis. In the *joint effortful* condition, objects started at two-fifths of the between-goal distance from the joint goal. On *equal effort* trials, objects started at one-third of the between-goal distance from the joint goal. Finally, on *solo effortful* trials, objects started at two-sevenths of the between-goal distance from the joint goal. Object locations varied slightly within each condition – appearing either at the above-described locations or with an offset to either side on the x-axis of 1% of the between-goal distance. The side on which the joint and solo goals appeared varied randomly between trials.

The combination of conditions (4), offsets (3), trial leader (2) and goal-side (2) resulted in a total of 48 unique trials. These trials appeared once per block for four blocks, for a total of 192 trials. The experiment began with four practice trials.

## Results and discussion

To test whether participants’ goal choices differed between conditions, we fit a binomial mixed effects model on trial-level data with Condition as a fixed effect and random intercepts for participants. We found a significant main effect of Condition, indicating that participants’ likelihood of choosing the joint goal varied according to its distance relative to the solo goal, χ^2^(3) = 39.3, *p* < .001. We also attempted to fit models with random slopes, but these produced convergence issues in each of our experiments. Models with random intercepts for dyads produced near-identical results, so we do not report them here.

**Fig. 3:**
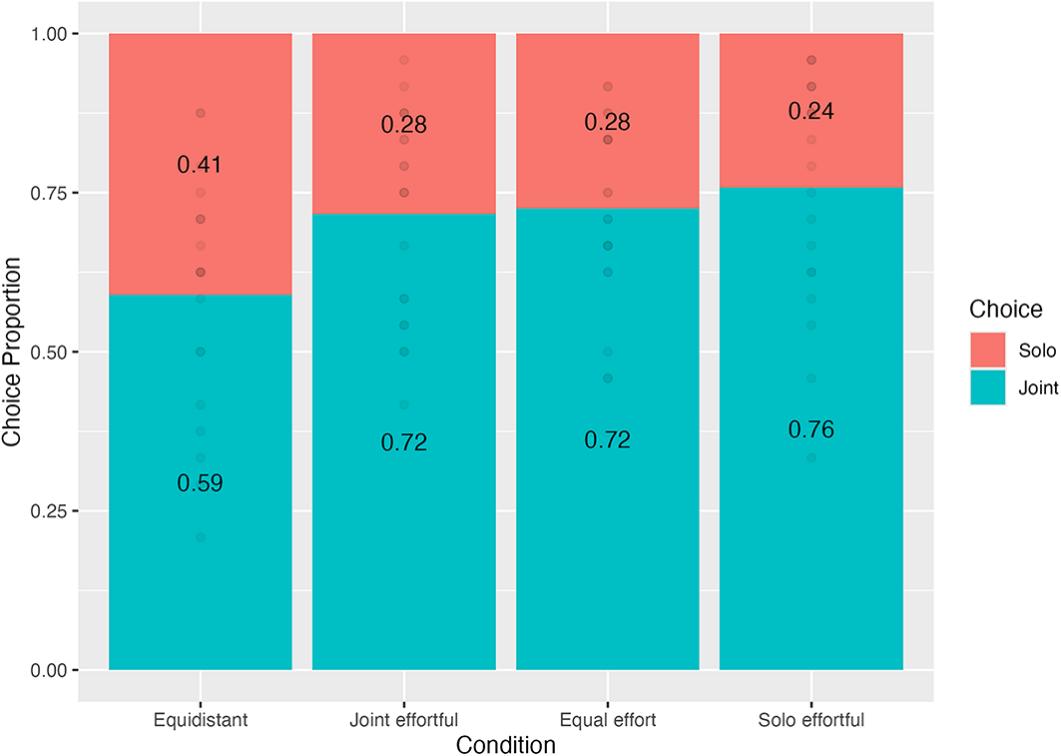
Choice proportions by Condition in Experiment 1.

Bonferroni-corrected post-hoc contrasts (Fig. 3) comparing log odds ratios (LRs) between conditions revealed that, compared to the equidistant condition, participants were significantly more likely to choose the joint goal in the equal effort (*z* = 4.56, LR_diff_ = − 0.651, 95%CI = [-1.02, − 0.284], *p* < .001), joint effortful (*z* = 4.27, LR_diff_ = − 0.606, 95%CI = [0.241, 0.972], *p* < .001), and solo effortful (*z* = 5.72, LR_diff_ = 0.834, 95%CI = [0.460, 1.21], *p* < .001) conditions. We did not find a difference in goal choice between the latter three conditions (*z*s ≤ 1.51, *p*s ≥ 0.78). Together, results imply that participants did not consider the overall cost differences between goals when making a decision, instead choosing the closer goal perhaps to minimise their time-on-task.

**Fig. 4:**
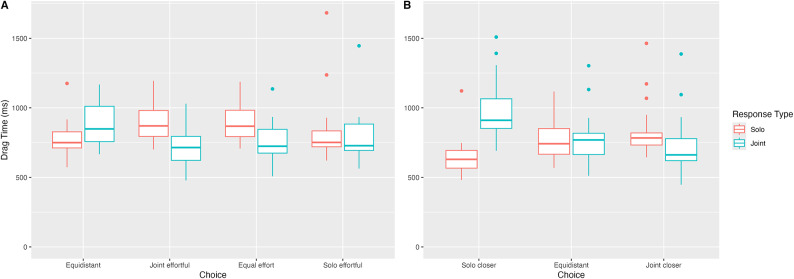
Drag Times by Condition in Experiments 1 (panel A) and 2 (panel B).

We analysed drag times to examine whether joint goals were indeed faster to reach (Fig. 4A). For solo goals, drag time was the time it took participants to drag the object into the goal after first touching it. For joint goals, we took the average of both participants’ solo drag times. We excluded drag times more than 3 standard deviations from condition means from analysis. A (2)(Goal: Solo vs. Joint) x (4)(Condition: Equidistant, Joint effortful, Equal effort, Solo effortful) ANOVA on drag times revealed neither a main effect of Goal nor for Condition (*p*s > 39). We did, however, find a Goal x Condition interaction *F*(1.74, 27.8) = 13.9, *p* < .001. Bonferroni-corrected pairwise comparisons revealed drag times to joint goals were faster in the joint effortful (by 200ms, *p* < .001) and equal effort (by 141ms, *p* < .001) conditions, but slower in the equidistant condition (by 112ms, *p* < .01). We did not find a difference between drag times in the solo effortful condition, *p* = .234.

Our results raise the question: if participants were making decisions based on distance, then why did we not find a difference between the three non-equidistant conditions? A possible explanation is that participants could not detect the differences in distances between the conditions. This is unlikely the case, considering that the difference in distance between the equidistant and joint effortful conditions – where we did find an effect - was smaller than that between the joint effortful and solo effortful conditions. Instead, it is likely that participants’ apparent preference for closer goals reached its ceiling at even the smallest asymmetry in goal distances. Interestingly, participants chose the joint goal at above chance level (Joint choice proportions ≥ 0.59, *p*s ≤ 0.03) in all four conditions. This tendency likely reflects a joint action preference, considering that we observed it even in the equidistant condition, where the joint goal was the same distance but required twice the dragging. This bias toward joint goals aligns with previous work, showing that people will exert more effort to perform the same task together rather than alone^11^. It is unlikely that the observed joint action preference masked or distorted other effects. Whatever the exact magnitude of the joint action preference is, it should remain the same across conditions.

In sum, by disentangling time and overall effort costs, Experiment 1 provides first evidence that people choose to minimise time-on-task, even if it requires more overall effort from the dyad. In addition, it seems that a pervasive preference for acting together biases decisions related to minimizing time, because participants in the equidistant condition showed a preference for joint action.

### Experiment 2

An alternative explanation for our results in Experiment 1 comes from the fact that the joint goal in the non-equidistant conditions always required less effort for the trial leader. So, it is possible that trial leaders simply sought to minimise their own effort by choosing the joint goal when it was closer. In Experiment 2, we aimed to test this possibility by varying whether solo dragging would be completed by the trial leader or their partner. In other words, half of the solo trials were as in Experiment 1- if the trial leader chose the solo option, they would drag one object to the goal. In the remaining solo trials, if the trial leader chose the solo option, their partner alone would need to drag one object to the goal. If participants decide to minimise their own effort, they should be more likely to choose the solo option when it would require their partner to act, thus absolving themselves of the need to act. Otherwise, if participants sought to minimise time, their choices should not change according to who is responsible for the solo action.

Additionally, we made changes to the object starting points. We removed the equal effort condition and instead added two starting points that mirrored the solo effortful and joint effortful starting points but on the side of the solo goal. We added these starting points to rule out the possibility that the closer-goal preference in Experiment 1 was contingent upon the goal being achieved jointly.

## Method

### Participants

We based power simulations for Experiment 2 on data from the equidistant, solo effortful and joint effortful conditions of Experiment 1. Simulations revealed that we needed just ten participants to detect a between-condition effect with power = 0.90 and alpha = 0.008 (adjusted for multiple comparisons). To ensure that we could detect an effect of whether solo goals required the leader or their partner to act, we recruited a larger sample of 24 participants (18 female, 6 male, M_age_ = 25.6, SD_age_ = 4.3, 18 right-handed, 6 left-handed). We discarded the data of one pair due to a technical error impacting stimulus display during their session and recruited an additional pair in their place.

### Procedure

The task procedure was identical to Experiment 1 with the following changes. In half of the trials, the solo action option would require the trial leader’s partner to drag an object. This was indicated in the solo option button, which was labelled ‘Alone (self)’ if the leader would need to act, but ‘Alone (partner)’ if their partner would. Rather than varying between trials, one participant remained the trial leader for the first half of the session and switched in the second. We did not inform participants that trial leaders would switch. This was to prevent the person first assigned to be the trial leader from considering reciprocity when choosing a goal.

**Fig. 5:**
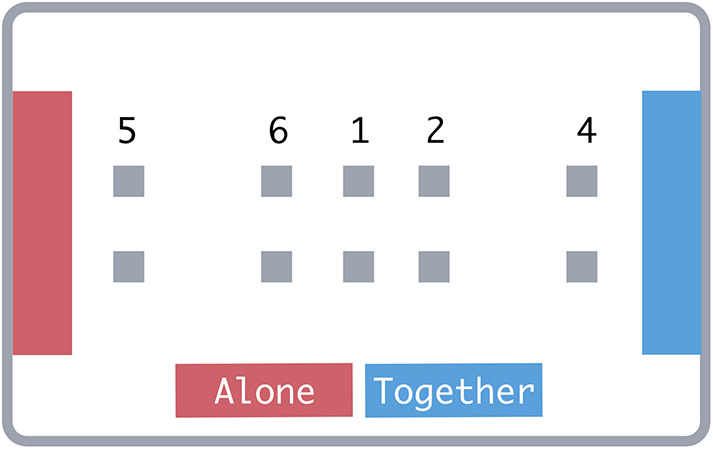
Object starting points in Experiments 2–3 numbered according to their entries in Table 1. (1) Equidistant condition, (2,4) Joint-closer conditions, (5–6) Solo-closer conditions. Display elements not to scale.

Trial structure was otherwise the same in Experiment 2, although there were changes in the objects’ starting points (see Fig. 5). We removed equal effort trials since participants in Experiment 1 were not sensitive to the effort differences in the three conditions closer to the joint goal. We kept the joint effortful and solo effortful conditions to keep variability in object locations. The two new starting point conditions were two-sevenths and two-fifths of the between-goal distance away from the solo goal – mirroring the conditions retained from Experiment 1 (see Fig. 2). Trials appeared in these four conditions with equal probability but twice as often in the equidistant condition so that it was equiprobable that objects appeared in the centre, closer to the solo goal or closer to the joint goal. Each participant completed one block of 72 trials as the trial leader. Participants completed four practice trials before each block.

## Results and discussion

Since we did not find a difference between non-equidistant conditions in Experiment 1, we collapsed them in Experiment 2 for a total of three conditions: equidistant, solo-closer, and joint-closer. We tested whether there were any differences between any of the collapsed conditions across Experiments 2 and 3. These tests did not reveal any significant differences (*ps* > 0.42).

**Fig. 6:**
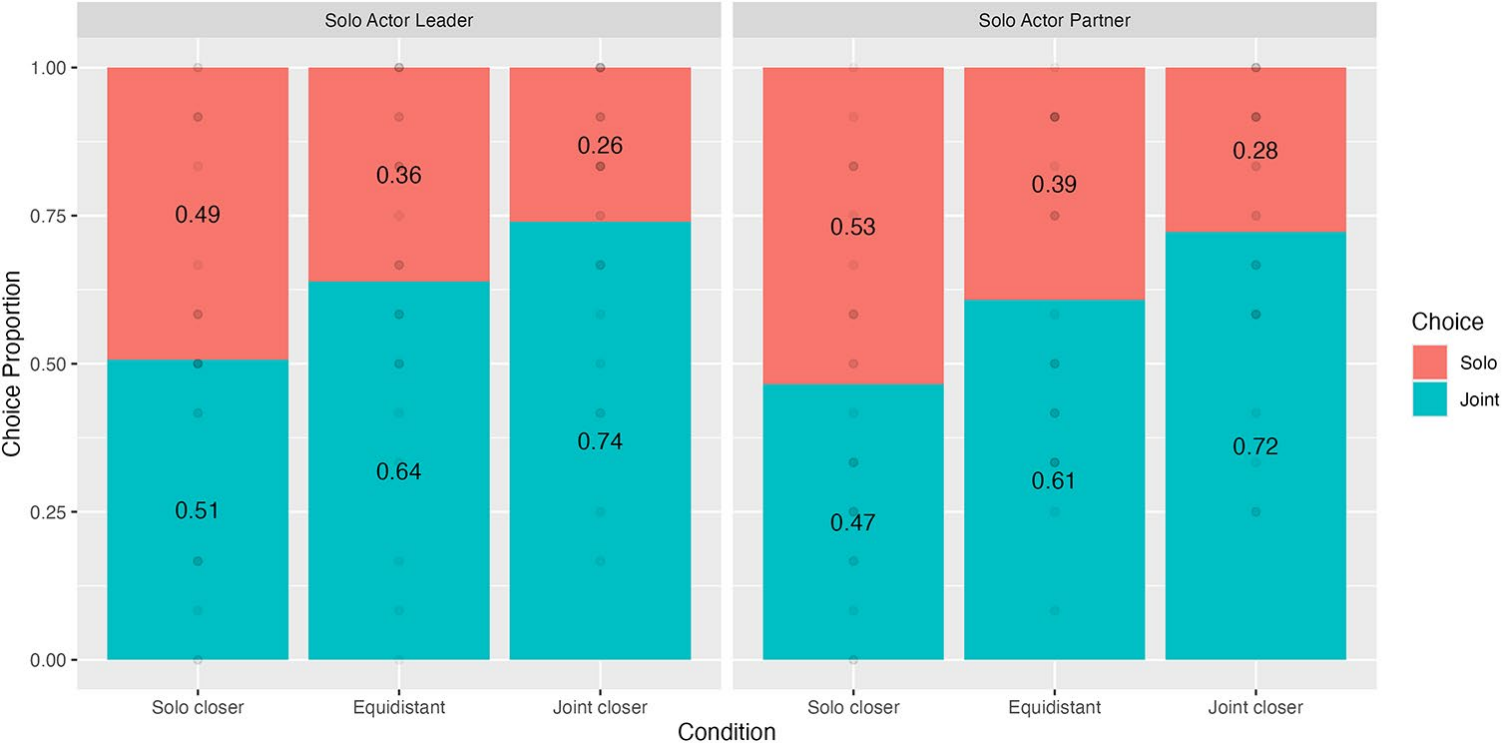
Choice proportions by condition in Experiment 2.

We fit a mixed-effects model as in Experiment 1 but with Solo Actor and Condition as interacting fixed factors. Again, we found a significant main effect for Condition, χ^2^(2) = 84.3, *p* < .001 (Fig. 6). Bonferroni-corrected post-hoc comparisons revealed that participants were more likely to choose the joint goal in the joint-closer (*z* = 6.79, LR_diff_ = 1.33, 95%CI = [0.869, 1.78], *p* < .001) and equidistant (*z* = 3.78, LR_diff_ = 0.709, 95%CI = [0.270, 1.15], *p* < .001) conditions than in the solo-closer condition. Further, the proportion of joint goal choices was higher in the joint-closer vs. equidistant condition (*z* = 3.18, LR_diff_ = 0.618, 95%CI = [0.162, 1.07], *p* < .01).

Critically, we found neither a main effect of Solo Actor (χ^2^(1) = 2.09, *p* = .15) nor an interaction between the two factors, χ^2^(2) = 0.142, *p* = .93. In other words, participants did not seem to choose joint goals merely to minimise their own effort. Instead, Experiment 2 shows that participants favour closer goals even when they require solo action or more effort from either individual.

As in Experiment 1, we found evidence for a joint action preference – participants chose the joint goal at above chance in the equidistant and joint-closer conditions (Proportion of joint choices ≥ 0.62, *p*s ≤ 0.02), but not in the solo-closer condition (0.49, *p* = .84). We note that the latter does not imply there was no bias toward joint action in the solo-closer condition, since participants chose a goal at around chance whereas we would expect them to choose the solo goal more often if they minimised distance or time alone. While previous work has shown a joint action preference when a solo actor recruits an otherwise unoccupied partner^11^, we show that this preference remains even when one can volunteer to help an otherwise solo actor.

Expectedly, we found a significant Goal x Condition interaction on drag times, *F*(1.57, 28.3) = 26.3, *p* < .001; Fig. 4B). Bonferroni-corrected contrasts revealed that drag times to joint goals were faster in the joint closer condition (by 148ms, *p* < .01) and slower in the solo closer condition (by 278ms, *p* < .001). We did not find an effect of Goal in the equidistant condition, *p* = .479.

### Experiment 3

In Experiment 3, we tested whether participants choosing goals that required less dragging indeed reflected a strategy to minimise time costs or if participants made decisions using distance as a heuristic. We changed the response mode so that participants only single-tapped the screen to instantly ‘teleport’ objects to their goals. This meant that neither time nor effort costs were associated with the distance between the objects and each goal. In Experiment 3a, participants single-tapped the objects to teleport them to the goal. In Experiment 3b, participants instead tapped the green ‘Go’ indicator, which appeared in front of them after choosing a goal (see Fig. 1, action phase) to move the objects. This enabled us to test whether the effects in Experiment 3a required attention to relative goal distances since participants in 3b would not need to interact with the objects at all. We expected that if participants chose closer goals via a distance heuristic rather than minimising action costs, they would still favour closer goals even when goal distance was dissociated from time and effort.

## Method

### Participants

Power simulations based on Experiment 2 indicated that we needed a sample of five participants in each experiment to achieve power = 0.90 with alpha = 0.016. We considered it possible that both time-minimisation and heuristic strategies contributed to effects in Experiments 1 and 2. We therefore anticipated that the condition effects in Experiments 3a and 3b could be smaller. To account for this, we recruited a larger sample of 20 participants for each Experiment (3a: 12 female, 8 male, M_age_ = 25.2 SD_age_ = 4.12, 16 right-handed, 4 left-handed; 3b: 15 female, 5 male, 1 other, M_age_ = 25.8, SD_age_ = 4.74), 19 right-handed, 1 ambidextrous).

### Procedure

The task structure for both experiments was identical to Experiment 2 with the following exceptions. As in Experiment 1, the trial leader varied between trials, and choosing the solo goal only required the leader to act. In Experiment 3a, participants first tapped the ‘Go’ button and then responded by tapping the objects once, after which the corresponding object instantly moved to the chosen goal. We asked participants first to press ‘Go’ to control for the distance between the option and objects. In Experiment 3b, participants did not interact with objects directly but teleported them by tapping the ‘Go’ button. Since we expected trial times to be much shorter, we doubled the number of trials (288 across four blocks) for more precise effect estimates.

## Results and discussion

**Fig. 7:**
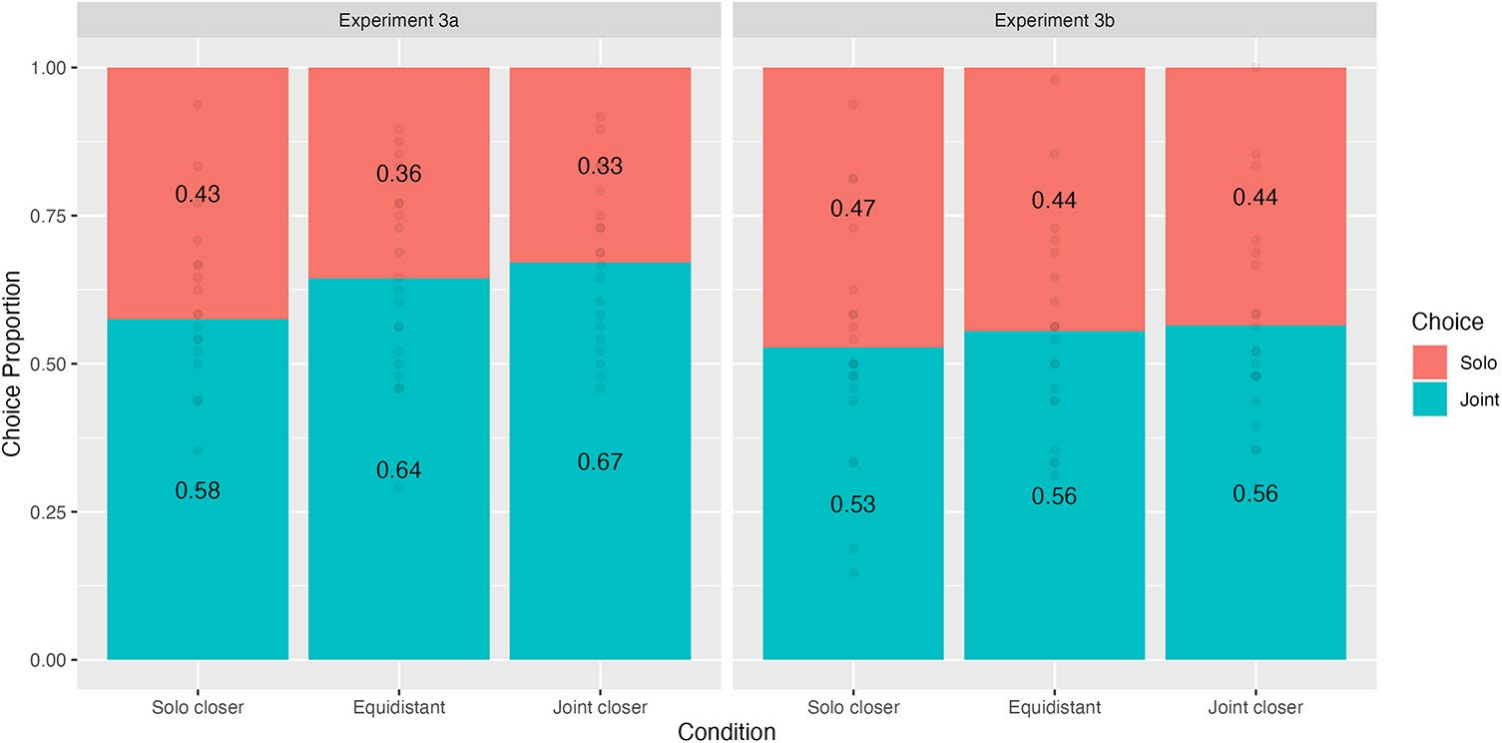
Choice proportions by Condition in Experiments 3a and 3b.

We found a main effect of Condition in Experiment 3a, χ^2^(2) = 21.7, *p* < .001 (Fig. 7). Bonferroni-corrected comparisons revealed that participants were more likely to choose the joint goal in the equidistant (*z* = 3.22, LR_diff_ = − 0.445, 95%CI = − 0.544, − 0.086], *p* < .001) and joint-closer conditions (*z* = 4.51, LR_diff_ = − 0.315, 95%CI = − 0.677, − 0.214], *p* < .001) compared to the solo-closer condition. We did not find a difference between the equidistant and joint-closer conditions, *z* = 1.30, LR_diff_ = 0.130, 95%CI = [-0.104, 0.365], *p* = .579. The main effect of Condition was not significant in Experiment 3b, χ^2^(2) = 3.34, *p* = .18.

Together, these results imply that participants only applied the distance heuristic to their decision-making when the relative distances between the object and goals were more salient (i.e. when participants had to tap the objects vs. tapping ‘Go’). This account also explains why the between-condition differences were less pronounced than those in Experiments 1 and 2, in which the distance cue was more salient due to it being relevant to participants’ subsequent actions.

To test for between-experiment differences, we also fit a model on the combined data of Experiments 3a and b with fixed effects for Condition, Experiment, and their interaction. This analysis only revealed a main effect of Condition, χ^2^(2) = 20.0, *p* < .001, other *ps* = 0.12. As in Experiment 3a, post-hoc tests showed that participants displayed a greater preference for the joint goal in the equidistant and joint-closer conditions compared to the solo-closer condition, *p*s < 0.001. We did not find a difference in choices between the joint-closer and equidistant conditions, *p* = .602. We note that our power simulations – and therefore sample sizes - were designed to assess power to detect a Condition effect within an experiment and not for a Condition x Experiment interaction, so we interpret the lack of a between-experiment effect cautiously.

We found a joint action preference in all three conditions in Experiment 3a (Proportion of joint choices ≥ 0.58, *p*s ≤ 0.01) but only in the joint-closer condition in Experiment 3b (Proportion of joint choices = 0.56, *p* = .02). The fact that in Experiment 3a people showed a preference for acting together even in the solo closer and in the equidistant condition indicates that this preference to some extent overrules distance cues.

## Discussion

Across three experiments, we investigated whether people try to minimise time or effort when working with a partner and to what extent people use rational decision-making or rely on heuristics. To this end, we varied the relative joint effort costs of goals by simultaneously manipulating goal distance and whether a goal required both participants to act in parallel. This enabled us to have conditions where the more effortful goal required less time to reach (see Table 1). Experiments 1 and 2 supported the prediction that participants would seek to minimise time rather than effort costs when choosing a goal. In Experiment 1, participants chose the closer (i.e., faster), joint, goal (in the non-equidistant conditions) at the same rate even when it required more overall effort from the dyad. The effect of goal distance persisted both when the closer goal required a solo action and when choosing the solo alternative required one’s partner to act rather than oneself (Experiment 2). These findings indicate that distance cues help actors to minimise the time spent on the task.

In Experiments 3a and 3b, we tested whether dyads minimised action costs via heuristics (in this case, distance) or via a rational decision-making process. In Experiment 3a,

participants showed a preference – albeit to a lesser extent than in Experiments 1 and 2 - for the closer goal even when the time costs were the same (and negligible) for either option. These results suggest that individuals rely on heuristics – in this case, the distance between an object and its goal – to minimise their action costs. Interestingly, this effect was contingent upon participants needing to interact with objects by tapping them and did not replicate when participants instead pressed a button to move them (Experiment 3b). One interpretation of this finding is that individuals rely more on cost-minimising cues – even when counterproductive - when costs become more salient. Since reaching and tapping required more time and effort than pressing a button, participants might have paid more attention to relevant cues vs. when costs were negligible. Further studies with larger sample sizes seem needed to address potential differences between interacting with objects versus controlling them more indirectly.

Taken together, our results demonstrate that people rely on distance as a heuristic cue to determine the course of their actions, be it acting alone or together. Importantly, the finding that participants did not minimise effort casts doubt on claims from previous studies that dyads seek to minimise efficiency at the group rather than individual level^6–8^. These studies have orthogonally manipulated efficiency for the dyad and individual without separating time costs for both participants. In other words, the individually- and co-efficient options with respect to time were always the same. Thus, if individuals indeed prioritised minimising time – as our results would suggest - it remains unknown whether they did so to minimise their own or the dyad’s time costs.

Future studies might sidestep the inherent confounds between distance, time, and effort altogether by employing alternative paradigms. Tasks involving exerting various forces (e.g., grip) could enable researchers to manipulate effort costs while holding time constant. Such a design would also offer more precise control over the effort participants exert. A drawback of our design is that it operates on the assumption that effort increases linearly with drag distance - without controlling the muscles and joints participants recruit to move objects, we cannot be sure that this is true. For instance, individual differences in arm span could have resulted in some participants merely moving their arms to drag objects, while others leaned their bodies as they found goals to be outside of their peripersonal space. Nevertheless, since participants chose goals based on distance even when effort costs were negligible, these additional effort-related variables likely would have had little impact on goal choice.

In line with previous work^11^, we found a clear preference for joint action when people had a choice between acting alone and together. Although distance predicted between-condition differences in goal choice, participants chose to act jointly well above chance except when the solo goal was closer - even when acting together implied covering a longer distance than acting alone. This shows that people’s decisions are not only driven by distance as a cue to action costs, as decisions are biased by our tendency to cooperate^12^ and coordinate our actions with others^11^.

A potentially pertinent difference between our study and prior studies on co-efficiency^6–8^ is that here, participants chose between acting alone and with their partners. In contrast, both action alternatives required joint action in prior studies. Due to this difference in design, an (irrational) preference for joint action could be demonstrated in our experiments. At the same time, this difference in design should have little bearing on the interpretation of the results concerning the use of distance as a heuristic cue. Just as distance served as a cue in the present experiments, it may also have guided participants’ decisions in earlier studies on co-efficiency. Our results, therefore, raise questions about how people decide on the course of joint actions and their individual contributions if they do not engage in rational decision-making. As we have shown, decisions are guided by a general preference to act together, combined with a heuristic to choose movement paths that minimise distance, which tends to minimise movement effort and time. The fact that people rely on distance cues even when distance does not minimise effort and/or time (Experiment 3a) indicates that people indeed use them as a proxy rather than computing the actual costs of their own and their co-actor’s potential movements.

It seems likely, though, that there are situations where people need to compute action costs in terms of the actual effort involved. In our study, the amount of time and effort required for either action alternative was small compared to many everyday actions. Perhaps people switch to a rational approach when there is more at stake, and the cost of choosing an inefficient action based on a heuristic is too high. Some joint tasks - like lifting and moving real-world objects - are especially effortful and might invite a rational approach to ensure we choose the most efficient path. Further, since costs for some actions depend on multiple variables – for lifting, the object’s size, shape, and surroundings – a single heuristic might accurately identify the most efficient option. An interesting question for future work on individual and joint action planning is how heuristics and more detailed cost computations – like minimising working memory load^13^—operate in concert.

Another open question is how people switch between cost-minimisation strategies when their resources are limited and how dyads might divide tasks when there is a resource asymmetry. It is well-established that people undergo a compensatory reduction in calorie expenditure following intense exercise or dietary restriction^14^. Future work might test how people attend to different cues depending on their resource limitations. Of particular interest to joint action research is how people distribute tasks when group members have different resource limitations. Do we assign faster-to-complete tasks to a partner who is in a rush? Or less effortful tasks to a partner who is tired?

In sum, our results indicate that when deciding how to act, dyads generally prefer joint action and rely on heuristics based on distance information rather than on more detailed computations of effort or time. More broadly, our findings highlight the importance of future research investigating how these heuristics are used in contexts where co-actors have unequal abilities or resources.

## Data Availability

The datasets analysed during the current study are available in the OSF repository, https://osf.io/nhvs5/.
